# Rapid detection of carbapenemases in multiresistant Gram-negative strains: evaluation of two tests

**DOI:** 10.1128/spectrum.02789-24

**Published:** 2025-09-10

**Authors:** Jennifer Schweer, Franziska Peters, Parviz Ahmad-Nejad

**Affiliations:** 1 Institute for Medical Laboratory Diagnostics, Helios University Hospital, Witten/Herdecke University 12263, Wuppertal, Germany; Ross University School of Veterinary Medicine, Basseterre, Saint Kitt and Nevis

**Keywords:** molecular diagnostics, carbapenemases, NAAT, rapid test

## Abstract

Carbapenem-resistant organisms (CRO) have rapidly spread worldwide in recent years, posing a significant challenge to both human health and healthcare systems. Timely and accurate detection of CRO, especially carbapenemase-producing *Enterobacterales* and non-fermenters, is crucial for clinical prevention and treatment of these infections. In the present study, we subjected more than 114 multidrug-resistant Gram-negative *Enterobacterales* and non-fermenters to two tests for the timely detection of carbapenemases. The strains were exclusively clinical isolates, and they were examined using both the CARBA-5 (NG Biotech) test and the molecular genetic analysis via the Allplex Entero-DR assay (Seegene). The NG CARBA-5 test exhibited a sensitivity of 63.2% (72/114), whereas the Allplex Entero-DR assay achieved a sensitivity of 71.9% (82/114). In both tests, it was primarily non-fermenters—*A. baumannii* (11/13 84.62%) and *P. aeruginosa* (11/21 52.38%)—in which the test did not detect any carbapenemase. Moreover, direct PCR-based detection of carbapenemases from the primary swab tube was successful in 12 out of 12 samples. This investigation highlighted that there is room for improvement in sensitivity when performing multiplex lateral flow immunochromatographic assays, as well as PCR-based methods. The direct detection of carbapenemases in primary swab tubes represents a broadly applicable approach for future molecular genetic testing methods.

**IMPORTANCE:**

Rapid and accurate detection of carbapenemase-producing bacteria is essential for infection control and effective treatment, especially as antimicrobial resistance continues to rise worldwide. Carbapenem-resistant Gram-negative pathogens, including *Enterobacterales* and non-fermenters, are particularly challenging due to limited therapeutic options and high transmission risk in healthcare settings. This study evaluates two diagnostic approaches—a lateral flow assay and a PCR-based molecular test—for detecting key carbapenemase genes. By comparing their performance in over 100 multidrug-resistant clinical isolates, the study provides practical insights into the sensitivity and limitations of each method. Importantly, it demonstrates that direct PCR testing from primary patient material is feasible and yields reliable results. These findings support the use of molecular diagnostics for early resistance detection and help inform clinical decision-making and infection control strategies. The results are especially relevant for microbiology laboratories aiming to improve diagnostic workflows and enhance preparedness against resistant pathogens.

## INTRODUCTION

Carbapenemases are enzymes that render bacteria resistant to carbapenems, a group of potent antibiotics. This resistance complicates the treatment of infections, as carbapenems are often used as a last resort for severe infections (1). The spread of such bacteria is a public health concern, as they can significantly undermine the effectiveness of antibiotics (2–4).

The prevalence of carbapenem-resistant organisms (CRO) worldwide varies depending on the region, healthcare system, antibiotic usage, and other factors. These strains can be found in hospitals, communities, and other environments. There is growing concern about the increasing global spread of antibiotic resistance (4–11). Among Gram-negative bacteria, resistance to carbapenems shows the greatest increase compared to other antibiotic classes. The number of deaths associated with this resistance rose from 619,000 in 1990 to 1.03 million in 2021. The number of deaths directly attributable to this resistance also increased significantly—from 127,000 to 216,000 over the same period (12). Carbapenemases belong to a group of enzymes known as β-lactamases, capable of cleaving the β-lactam ring structure of carbapenem antibiotics, leading to their inactivation. There are several classes of carbapenemases classified based on their genetic and structural properties. The five common carbapenemase classes are defined by the encoding genes *bla*_KPC_, *bla*_IMP_, *bla*_VIM_, *bla*_NDM_, and *bla*_OXA-48_ (13, 14).

The most important carbapenemases are as follows (15–17): KPC (*Klebsiella pneumoniae* carbapenemase) is a class A carbapenemase that was originally identified in *Klebsiella pneumoniae* bacteria. KPC enzymes have the ability to degrade both β-lactam antibiotics and carbapenems. OXA (oxacillinase) is a class D carbapenemase that was initially known for the degradation of penicillin antibiotics; however, some OXA enzymes are also capable of cleaving carbapenems (18–20). VIM (Verona integron-metallo-β-lactamase) is a class B carbapenemase, also referred to as a metallo-β-lactamase. VIM enzymes require metal ions, such as zinc, as cofactors to cleave carbapenems. IMP (imipenemase) is another class B carbapenemase or metallo-β-lactamase. Like VIM enzymes, IMP enzymes also require metal ions for the cleavage of carbapenems. NDM (New Delhi metallo-β-lactamase) is another class B carbapenemase, first identified in bacteria in India. It also belongs to the group of metallo-β-lactamases and is known for its ability to degrade carbapenems (17, 21).

The precise classification and identification of these enzymes are pivotal to developing appropriate therapeutic approaches and controlling the spread of antibiotic resistance. Rapid detection and hygiene-related measures are increasingly important in the care of critically ill patients. Diagnostic assays for the detection and confirmation of carbapenemases are essential for the early identification of these resistance mechanisms and for the effective containment of carbapenem-resistant bacteria in contemporary hospital-associated healthcare environments. To address this issue, we conducted a comparative study on the detection of resistance genes in carbapenem-resistant organisms (CRO) in the present work. We compared the detection using immunochromatographic rapid tests, NG CARBA-5 test (NG Biotech), and multiplex real-time PCR Allplex Entero-DR (Seegene).

## MATERIALS AND METHODS

The present study was approved by the Ethics Committee of the University of Witten/Herdecke (S-155/2024).

The NG CARBA-5 test (NG Biotech, France) detects the carbapenemases KPC, OXA-48-like, VIM, IMP, and NDM from carbapenem-susceptible bacterial colonies of *Enterobacterales* (*Escherichia coli* and *Klebsiella pneumoniae*) and *Pseudomonas aeruginosa*. A pure colony from the bacterial culture is required for this purpose. The detection limits are 600 pg/mL for KPC, 300 pg/mL for OXA and VIM, 200 pg/mL for IMP, and 150 pg/mL for NDM. The CARBA-5 test detects the following NDM types:

- NDM: NDM-1, -2, -3, -4, -5, -6, -7, -8, -9, -11, and -19
- KPC: KPC-1, -2, -3, -4, -5, -6, -7, -12, -14, -23, -28, and -39
- IMP: IMP-1, -2, -3, -4, -5, -6, -7, -8, -10, -11, -13, -14, -15, -16, -18, -19, -22, -26, -29, -31, -37, -39, -46, -47, -56, -58, -61, -71, and -79
- VIM: VIM-1, -2, -4, -5, -6, -19, -23, -26, -27, -31, -39, -46, -51, -52, -54, -56, -58, and -59
- OXA-48-like: OXA-48, -162, -181, -204, -232, -244, -245, -370, -436, -484, -515, -517, -519, -535, and -793.

The Allplex Entero-DR assay (Seegene, Seoul, Korea) is a qualitative *in vitro* diagnostic test that utilizes multiplex real-time polymerase chain reaction (PCR). This test enables the simple or simultaneous detection of carbapenemase genes (NDM, KPC, OXA-48, VIM, and IMP), extended-spectrum beta-lactamase (ESBL) gene (CTX-M), and vancomycin resistance genes (VanA and VanB) from rectal swabs and bacterial colonies. The detection limit for the Allplex Entero-DR assay is 100 copies/reaction. The detection of VanA and VanB in Gram-positive bacteria is possible by the test, but this is not part of the present study and will therefore not be discussed further.

For genotyping, the Entero-DR Assay from Seegene was used, following the manufacturer’s recommendation. A total of 5–10 colonies were used for DNA isolation and supplemented with 10 µL kit-specific internal control (IC). The extraction was performed using the STARMag 96 × 4 Universal Cartridge Kit (Seegene) on the Nimbus platform (Seegene). Isolated DNA samples were used in the Allplex Entero-DR assay (5 µL per reaction) and mixed with PCR reaction mix according to the manufacturer’s instructions. The thermal cycling and target detection were performed using a Bio-Rad CFX96 real-time PCR instrument (Bio-Rad Laboratories Inc., San Francisco, USA). The results were analyzed using the Seegene Viewer software (version 3.30.000).

If primary swab tubes (eSwab, COPAN, Italy) were directly subjected to PCR analysis, 200 µL of material was used for DNA isolation.

### Characterization of the study cohort

Samples requiring pathogen or resistance screening were routinely cultured in the laboratory on selective ESBL media or Columbia universal plates. A total of 114 microbial strains were identified by Matrix-Assisted Laser Desorption/Ionization Time-of-Flight mass spectrometry (MALDI-TOF MS) (Bruker, Bremen, Germany) and tested for resistance to ertapenem, imipenem, and meropenem using agar diffusion, Phoenix (Becton Dickinson, Heidelberg, Germany), or Etest (Bestbion, Germany). Strains were therefore selected based on their *in vitro* resistance antibiotic pattern. We did not include isolates that were tested carbapenem-susceptible in our data set; therefore, it is not possible to compute specificity, PPV, or NPV in a statistically meaningful manner.

The study isolates include microorganisms from the last three years (2021–2023). An overview of the microorganisms examined in this study is provided in Table 1. The study included all strains that showed carbapenem resistance in antimicrobial susceptibility testing according to EUCAST. We acknowledge that this may also include strains that do not express a carbapenemase but also display multidrug efflux pump, porin loss, and a combination of these mechanisms along with another non-carbapenemase beta-lactamase (such as ampC). Nevertheless, we considered this to be the best approximation of the practical microbiological diagnostic procedure.

**TABLE 1:** Distribution of the examined bacterial strains

Microorganism	Number
*A. baumannii*	13
*A. pittii*	1
*C. freundii*	5
*E. bugandensis*	1
*E. cloacae*	14
*E. coli*	10
*K. aerogenes*	7
*K. pneumoniae*	41
*P. aeruginosa*	21
*S. marcescens*	1
Total	114

## RESULTS

### Test performance

The CARBA-5 test detected carbapenemase in 72 out of 114 examined Gram-negative bacteria, achieving a sensitivity of 63.2%. OXA-48 and NDM carbapenemases were by far the most commonly detected carbapenemases through immunochromatographic testing, with 41 and 26 detections, respectively. Molecular genetic detection using the Allplex Entero-DR Assay succeeded in *n* = 82/114 cases, yielding a sensitivity of 71.9%. The difference in sensitivity between the two tests was statistically significant (*P* = 0.016; McNemar’s test χ² = 5.82). Similar to the NG CARBA-5 assay, OXA-48 and NDM carbapenemases were the most frequently detected, with 44 and 31 detections, respectively (see Table 2). Imipenemase (IMP) was not detected in any case. The PCR was able to detect a total of six additional NDM, three VIM, and four OXA-48 carbapenemases in the examined Gram-negative bacteria.

**TABLE 2:** Number and percentage distribution of different carbapenemases within the examined samples (*n* = 114) using the NG CARBA-5 test and Seegene’s Allplex Entero-DR-PCR assay

NG CARBA-5	NDM	KPC	OXA-48	VIM	IMP
Pos	26	8	41	10	0
Neg	88	106	73	104	114
Total	114	114	114	114	114
Allplex Entero-DR assay	NDM	KPC	OXA-48	VIM	IMP
Pos	31	8	44	13	0
Neg	83	106	70	101	114
Total	114	114	114	114	114

Performing the NG CARBA-5 test and the Allplex Entero-DR assay, no detection of carbapenemase was achieved for 42 and 32 microorganisms, respectively. In both tests, it is primarily the non-fermenters *A. baumannii* and *P. aeruginosa* that show negative test results (see Table 3).

**TABLE 3:** Number and listing of microorganisms that showed no detection of any carbapenemase in the NG CARBA-5 or PCR test

The number of microorganisms that tested negative	NG CARBA-5 test	Allplex Entero-DR assay
*A. baumannii*	12/13	11/13
*C. freundii*	1/5	0/5
*E. cloacae*	3/14	2/14
*K. aerogenes*	5/7	5/7
*K. pneumoniae*	5/41	3/41
*P. aeruginosa*	16/21	11/21
Total	42/114	32/114

Discrepant results were observed for a total of 11 strains. In nine out of 11 cases, PCR detected carbapenemases more frequently than the immunochromatographic test. In eight strains, PCR was able to detect the carbapenemase alone, while the CARBA-5 test yielded no positive results. Table 4 illustrates that this was particularly the case for *P. aeruginosa* (*n* = 5), but *Enterobacterales* were also found in this group. Only in the case of one *K. pneumoniae* strain was detection successful in the CARBA-5 test but not in the PCR.

**TABLE 4:** Representation of the disparate findings between the NG CARBA-5 test and the molecular genetic detection via the Allplex Entero-DR test

Pathogen	Result NG CARBA-5	Result Seegene Allplex Entero-DR assay
*P. aeruginosa*	Neg	NDM
*P. aeruginosa*	Neg	NDM
*E. bugadiensis*	KPC	NDM, KPC
*P. aeruginosa*	Neg	NDM, OXA-48
*P. aeruginosa*	Neg	NDM, OXA-48
*A. baumannii*	Neg	NDM
*K. pneumoniae*	OXA-48	Neg
*K. pneumoniae*	NDM, OXA-48	OXA-48
*E. cloacae*	Neg	OXA-48, VIM
*C. freundii*	Neg	OXA-48, VIM
*P. aeruginosa*	Neg	VIM

We were interested in which combinations of carbapenem-hydrolyzing enzymes might be present in the individual Gram-negative bacteria, as a single bacterium can carry multiple different enzymes. The molecular genetic test was able to detect multiple combinations here as well, more than the CARBA-5 test.

In five microorganisms, the molecular genetic test was able to detect combinations of carbapenemases that were not detected by the immunochromatographic test. Overall, the proportion of detections of multiple carbapenemases per microorganism using the CARBA-5 test was 13/72 (18.1%), and with the PCR test, it was 17/82 (20.7%) (see Table 5).

**TABLE 5:** The number of detected genotype combinations in the NG CARBA-5 and Allplex PCR tests

Genotype combinations	NG CARBA-5	Allplex Entero-DR assay
KPC	8	7
NDM	13	16
NDM, OXA-48	13	14
NDM, KPC	0	1
OXA-48	28	28
OXA-48, VIM	0	2
VIM	10	11
Neg	42	35
Total	114	114

In addition to the aforementioned carbapenemases, the Allplex Entero-DR assay allows for simultaneous molecular detection of CTX-M beta-lactamases. CTX-M beta-lactamases are a genetically related group of extended-spectrum beta-lactamases (ESBLs). They belong to the serine protease class A according to Ambler’s classification. The name CTX-M is an acronym, where CTX stands for resistance to cefotaxime (CTX), and M refers to Munich, the place of first isolation. Although CTX-M is not a carbapenemase, its presence in multiresistant pathogens significantly complicates therapy. Therefore, its identification in clinical isolates is essential for resistance management and infection control. In our study, CTX-M was detected in 40.4% (*n* = 46/114) of the microbes investigated. Table 5 presents the identified combinations of resistance genes. In total, 13 strains had NDM, OXA-48, and CTX-M; 16 strains had OXA-48 and CTX-M; and in individual cases, there were KPC and CTX-M, OXA-48, CTX-M, and VIM, or CTX-M and VIM. In three cases, only the presence of CTX-M was confirmed (see Table 6).

**TABLE 6:** The number of detected genotype combinations in Allplex PCR tests including CTX-M detections

Genotype combinations	Number
NDM, CTX-M	8
NDM, KPC, CTX-M	1
NDM, OXA-48	1
NDM, OXA-48, CTX-M	13
CTX-M	3
CTX-M, VIM	1
KPC	6
KPC, CTX-M	1
NDM	8
OXA-48	12
OXA-48, CTX-M	16
OXA-48, CTX-M, VIM	1
OXA-48, VIM	1
VIM	10
Neg	32
Total	114

### Direct PCR testing from the primary swab tubes

To determine whether PCR is feasible directly from the primary material, we compared the direct application of the Allplex Entero-DR assay on the original sample (swab) and from the culture in 10 patients. The original samples included anal swabs (*n* = 3), a nasopharyngeal swab (*n* = 1), a urine sample (*n* = 1), tracheal secretion (*n* = 1), bronchial secretion (*n* = 1), pleural swab (*n* = 1), vaginal swab (*n* = 1), and blood culture (*n* = 1)(see Table 7). The PCR results from the culture confirmed the findings of the CARBA-5 test. The PCR conducted on the original material completely matched the PCR performed on the culture for the parameters NDM, KPC, OXA-48, and VIM.

**TABLE 7:** Comparison of the direct application of the Allplex Entero-DR assay on the original sample (e.g., eSwab) and from the cultured multiresistant strains in 10 patients

Method of detection	Sample type	NDM	KPC	OXA-48	VIM	IMP	CTX-M
CARBA-5 test	Culture	4	1	7	0	0	-
Allplex Entero-DR assay	Culture	4	1	7	0	0	7
Allplex Entero-DR assay	Initial sample	4	1	7	0	0	4
Initial sample PCR confirms culture PCR		4/4	1/1	7/7	0/0	0/0	4/7
		100%	100%	100%	100%	100%	57%

## DISCUSSION

The increasing incidence and prevalence of multidrug-resistant Gram-negative rod bacteria pose a threat to the healthcare system and present healthcare providers with limited treatment options for infections (12, 22). By implementing evidence-based measures for infection prevention and transmission control that consider transmission routes and pathogen biology, further spread can be curtailed. A key measure is the establishment of a rapid and accurate monitoring system capable of uncovering various genetic resistance mechanisms, ultimately leading to an effective surveillance. In the fight against multidrug-resistant organisms, in addition to antibiotic stewardship programs, enhanced hygiene, and mass media campaigns, the use of rapid diagnostic tests is a part of an intervention package (2, 23, 24).

For the rapid diagnosis of carbapenemases and for the confirmation of primary findings, several test methods are now available commercially. In addition to detection methods based on molecular genetic assays in LAMP (loop-mediated isothermal amplification) (25) or PCR cartridge formats or involving complex next-generation sequencing (26), there are also immunochromatographic and molecular genetic approaches available (26–30). While immunochromatographic rapid test methods require only a few clones of a pure culture and can be performed on-site quickly without specific training, PCR-based methods for carbapenemase detection are much more complex, requiring specialized equipment and trained personnel (30). The data presented here relate to the performance of the CARBA-5 assay and the Allplex Entero-DR assay for the detection of carbapenemase enzymes.

In the present study, we aimed to clarify which of these diagnostic methods actually yields better results. We subjected 114 multidrug-resistant Gram-negative rod bacteria to both testing approaches. Regarding sensitivity, the CARBA-5 test showed a sensitivity of 63.2%, while the Allplex Entero-DR assay showed a sensitivity of 71.9%. This shows that the molecular genetic approach (PCR) has a slightly higher sensitivity in the detection of carbapenemases.

In 36.8% (*n* = 42/114) of the tested strains, no resistance gene could be detected using the immunochromatographic method. In contrast, the PCR method had a percentage of 28.1% (*n* = 32/114).

A notable proportion of phenotypically carbapenem-resistant isolates yielded negative results in both the CARBA-5 and Allplex Entero-DR assays, suggesting the absence of detectable carbapenemase genes by the employed methods. This discrepancy may be explained by alternative resistance mechanisms not targeted by both assays. Specifically, overexpression of efflux pumps, reduced outer membrane permeability due to porin loss or mutations, or upregulation of AmpC β-lactamases in combination with permeability defects are well-documented non-carbapenemase-mediated mechanisms of carbapenem resistance. These mechanisms may result in false-negative outcomes in both phenotypic and genotypic carbapenemase detection assays. Future studies incorporating whole-genome sequencing or targeted analysis of efflux and porin gene expression could help elucidate the underlying resistance determinants in such cases.

Among the carbapenemases, OXA-48 and NDM were the most frequently detected carbapenemases in both tests. This reflects the importance of these carbapenemases in clinical settings. Discrepancies between test results were observed in 11 cases. In nine of these cases, the PCR was able to detect carbapenemases more frequently than the CARBA-5 test. The molecular genetic test method was able to detect more combinations of carbapenemases than the CARBA-5 test. This indicates that PCR enables a more comprehensive identification of enzymes. The Allplex Entero-DR assay also enabled the simultaneous detection of CTX-M beta-lactamases that were detected in 40.4% of the microbes analyzed.

A significant advantage of PCR methods was their feasibility using primary swab material, and they provided consistent results in smaller study groups. This aspect should and will likely be further investigated in larger studies, especially considering that approaches for faster screening, possibly through pooling strategies, could be feasible.

Limitations of the study are the limited sample size investigated and its single-center nature and the focus on multidrug-resistant organisms from a major healthcare facility in Germany. Different prevalence of other resistance mechanisms in various countries might yield differing results. Furthermore, the diagnostic tests used are only capable of detecting carbapenemase types that are already incorporated into their detection systems. Consequently, emerging variants of carbapenem resistance genes—which are known to evolve rapidly—may not be identified using the current diagnostic methods.

Some isolates exhibited discordant results between the NG CARBA-5 lateral flow assay and the Allplex Entero-DR PCR assay. As whole-genome sequencing (WGS) or Sanger sequencing was not performed in this study, we acknowledge the inability to definitively resolve these discrepancies or confirm the presence of uncommon or novel carbapenemase genes. This constitutes a methodological limitation. Confirmatory sequencing-based approaches should be considered in future studies to validate test performance, resolve inconsistent findings, and potentially identify rare or emerging resistance determinants not covered by current assay targets.

These results provide valuable insights into the performance of the two test methods for the detection of carbapenemases and other antibiotic resistance genes in Gram-negative bacteria. The choice of test method may be critical, particularly in clinical settings where rapid and accurate identification of resistance genes is required. Further research and validation are likely to be necessary to clarify the applicability and reliability of these tests in different situations.
